# First report of Salmonella 1,4,[5],12:i:- in free-ranging striped dolphins (*Stenella coeruleoalba*), Italy

**DOI:** 10.1038/s41598-019-42474-6

**Published:** 2019-04-15

**Authors:** C. Grattarola, S. Gallina, F. Giorda, A. Pautasso, M. Ballardini, B. Iulini, K. Varello, M. Goria, S. Peletto, L. Masoero, L. Serracca, A. Romano, A. Dondo, S. Zoppi, F. Garibaldi, F. E. Scaglione, L. Marsili, G. Di Guardo, A. A. Lettini, W. Mignone, A. Fernandez, C. Casalone

**Affiliations:** 1 Istituto Zooprofilattico Sperimentale del Piemonte, Liguria e Valle d’Aosta, Torino, 10154 Italy; 2 Institute of Animal Health, University of Las Palmas de Gran Canaria, Arucas, Las Palmas, 35416 Spain; 3 Department of Earth, Environmental and Life Sciences, University of Genoa, Genoa, 16132 Italy; 4 Department of Veterinary Sciences, University of Turin, Grugliasco, Turin, 10095 Italy; 5 Department of Physical, Earth and Environmental Sciences, University of Siena, Siena, 53100 Italy; 6 Faculty of Veterinary Medicine, University of Teramo, Teramo, 64100 Italy; 7 Reference Laboratory for Salmonella, Istituto Zooprofilattico Sperimentale delle Venezie, Legnaro, Padua, 35020 Italy

## Abstract

Between 2015 and the beginning of 2018 (January-March), 30 cetaceans were found stranded along the Ligurian Sea coast of Italy. Necropsies were performed in 22 cases and infectious diseases resulted the most common cause of death. Three striped dolphins, showed a severe coinfection involving the monophasic variant of *Salmonella* Typhimurium (*Salmonella* 1,4,[5],12:i:-). The isolates were characterized based on antimicrobial resistance, Multiple-Locus Variable-number tandem-repeat Analysis (MLVA) and whole-genome sequencing (WGS). All isolates demonstrated the same multidrug resistant genotype (ASSuT isolates), showed three different MLVA profiles, two of which closely related, and were identified as Sequence Type 34. Moreover, Single nucleotide polymorphisms (SNP) analysis confirmed strong correlations between two out of the three isolates. To our knowledge, *S*. 1,4,[5],12:i:-, one of the most common serovars in cases of human infection and food sources worldwide, has not previously been described in marine mammals, and reports of *Salmonella*-associated disease in free-ranging cetaceans are rare. These results highlight the role of cetaceans as sentinel species for zoonotic and terrestrial pathogens in the marine environment, suggest a potential risk for cetaceans and public health along the North Western Italian coastline and indicate cetaceans as a novel potential reservoir for one of the most widespread *Salmonella* serovars.

## Introduction

Many marine mammal species share the coastal environment with humans and may serve as efficient sentinels for infectious agents, including those with a zoonotic potential, thus becoming important indicators for public health-related issues^1^.

The Mediterranean basin represents the largest enclosed sea on earth and an important marine biodiversity hotspot, being surrounded by heavily populated and industrialized coastal areas, which makes the impact of anthropic activities proportionally stronger than in any other basins. Marine mammals from such regions, moreover, show high tissue concentrations of organochlorine (OC) xenobiotics^2,3^, increasing their susceptibility to other anthropogenic stressors.

**Figure 1:**
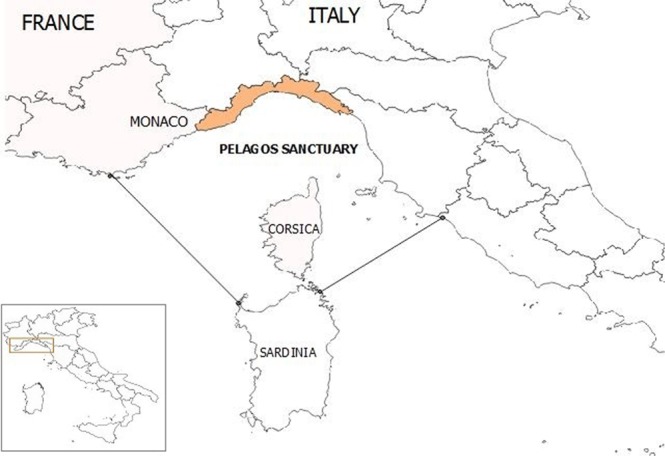
Map of the study area (Ligurian coastline) in the Pelagos Sanctuary area. The map was created by A.P. with QGIS (QGIS Development Team (2018). QGIS Geographic Information System. Open Source Geospatial Foundation Project. http://qgis.osgeo.org). Copyright © 2018 Alessandra Pautasso. Permission is granted to copy, distribute and/or modify this document under the terms of the GNU Free Documentation License, Version 1.3 or any later version published by the Free Software Foundation; with no Invariant Sections, no Front-Cover Texts, and no Back-Cover Texts.

Between 2015 and the beginning of 2018 (January-March), 30 cetaceans, of which 23 striped dolphins *(Stenella coeruleoalba)*, 2 bottlenose dolphins (*Tursiops truncatus*), 1 sperm whale (*Physeter macrocephalus*) and 4 undetermined specimens stranded along the Ligurian coast of Italy, in the Pelagos Sanctuary, a marine protected area for marine mammals covering about 90,000 km^2^ in the North Western Mediterranean basin, between Italy, Monaco and France, thereby encompassing the Northern coast of Sardinia, the Tuscan Archipelago and the entire Corsica Island (Fig. 1). Thanks to the surveillance activity of the Italian National Reference Centre for Diagnostic Activities on Stranded Marine Mammals (C.Re.Di.Ma.), necropsies were performed in 22 cases, the cause of stranding was determined with confidence in 19 cases and infectious diseases resulted the most common cause of death (18 out of 19). In half of the cases, the cetaceans under study were affected by severe coinfections involving cetacean-specific viruses, zoonotic pathogens and/or microbial agents indicative of environmental contamination.

**Figure 2:**
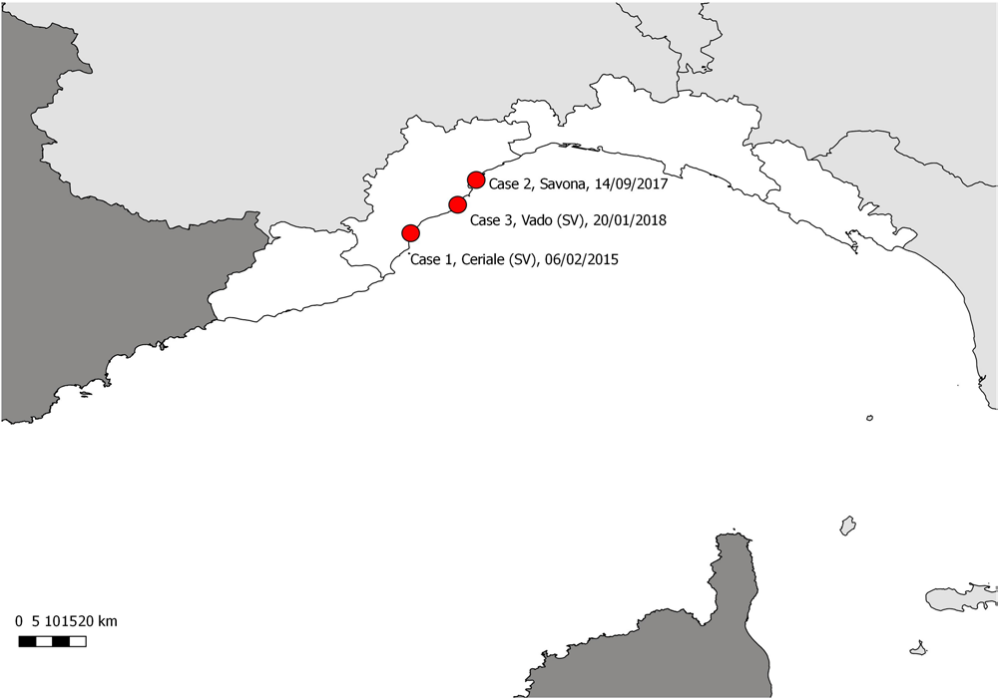
Map of the study area (Ligurian coastline), displaying the stranding locations of the 3 striped dolphins infected by *Salmonella* 1,4,[5],12:i:- variant (marked by red dots). The map was created by A.P. with QGIS (QGIS Development Team (2018). QGIS Geographic Information System. Open Source Geospatial Foundation Project. http://qgis.osgeo.org). Copyright © 2018 Alessandra Pautasso. Permission is granted to copy, distribute and/or modify this document under the terms of the GNU Free Documentation License, Version 1.3 or any later version published by the Free Software Foundation; with no Invariant Sections, no Front-Cover Texts, and no Back-Cover Texts.

Herein we report three cases of coinfection, involving striped dolphins found stranded along the coast of the province of Savona (Fig. 2), and characterized by the detection of a monophasic variant of *Salmonella* 1,4,[5],12:i:- in association with cetacean-specific viruses along with pathogens indicative of environmental contamination.

Although many marine species are known to harbor *Salmonella enterica*, reports of *Salmonella*-associated disease in free-ranging cetaceans are rare^4,5^, usually occurring in debilitated or stressed animals^6^. Clinically, severe enteritis with necrosis and/or haemorrhage are usually reported. Animals that develop septicaemia can die without showing clinical signs or, in some cases, can show complications such as bronchopneumonia, necrotizing hepatitis, splenitis, meningoencephalitis and abscessation^6,7^.

To our knowledge, the role of the environmental factors affecting *Salmonella* spp. persistence in the marine environment remains poorly understood, together with the infection’s transmission pathways to marine organisms. This faecal bacterium, in fact, is not indigenous to the marine environment, and its presence in coastal waters has been linked to heavy rain and storm-generated flows, transporting the contamination from their sources to the sea via river waters^8^, as well as to *in situ* defecation by infected marine animals^9^. Enteric bacteria may concentrate in sediments as well as in invertebrates and vertebrates of marine environments contaminated with faecal materials for prolonged periods. The presence of zooplankton and suspended marine particles colonized by *Salmonella* has been also reported, suggesting additional pathways for bacterial dissemination in marine habitats^10^.

Monophasic variant of *Salmonella* Typhimurium (*Salmonella* 1,4,[5],12:i:-) began to emerge in the mid-1990s and is currently one of the most common serovars in human clinical cases as well as in animal and food sources (mainly pigs and pork products) worldwide^11–15^, being responsible for a number of outbreaks, including those reported in Luxembourg, Germany, France and United States^16^.

In the last few years, in Italy, *S*. 1,4,[5],12:i:- has shown an increase in terms of prevalence, thereby ranking from the third most frequent *Salmonella* serovar isolated from clinical samples in 2008^17^ to the second in 2009^18^, and ultimately gaining the first position from 2011 onwards^19^.

This serovar exhibits the highest rate of multidrug resistance at the European level, thus representing a significant concern to public health authorities and also showing extremely high survival rates in the environment^20^. To the best of our knowledge, the monophasic variant of *Salmonella* Typhimurium has not been previously described in marine mammals.

In this study, three cases of coinfection, characterized by *S*. 1,4,[5],12:i:- detection, were analysed in detail, focusing on the pathogenic role of the identified agents, the association with the pathological findings and the most likely sources of exposure, considering the characteristics of the concerned coastline and the bacterial isolates’ features.

## Results

A summary of the results obtained is presented here below, with reference to necropsy, histopathological and analytical data for each case considered. Microbiological, virological and immunohistochemical results are also summarized in Table 1.

**Table 1:** Microbiological, virological and immunohistochemical results of investigations performed on the 3 striped dolphins under study.

Samples	Organs/Tissues	Investigations	Results	Reference
Case 1	**faeces, liver**	*Salmonella* spp. isolation	positive (*S*. 1,4,[5],12:i:-)	^38^
	**brain, lung**, PSC ln, TB ln, liver, **spleen**, kidney	standard aerobic bacterial culture	*Aeromonas hydrophila*	—
	brain	*Listeria* spp. isolation	negative	^38^
	brain, PSC ln, spleen	*Brucella* spp. isolation	negative	^38^
	brain, lung, PSC ln, spleen	*Brucella* spp. (PCR)	negative	^50^
	serum	anti*- Brucella* spp. AB detection (S.A.R.)	negative	^52^
	brain, **lung**, **PSC ln**, spleen	*Morbillivirus* (PCR, sequence analysis)	positive *(Dolphin Morbillivirus)*	^27^
	brain, lung, spleen, kidney	*Morbillivirus* (IHC)	negative	^25^
	lung, PSC ln	*Morbillivirus* (isolation)	negative	^51^
	serum	anti*-Morbillivirus* AB detection (SN)	negative	^53^
	**brain**, PSC ln, liver, spleen, muscle	*Toxoplasma gondii* (PCR)	positive	^26^
	brain	*Toxoplasma gondii* (IHC)	negative	^25^
	**serum**	*anti-T. gondii* AB detection (IFAT)	positive (>1:160)	^25^
	brain, lung, PSC ln, spleen	*Herpesvirus* (PCR)	negative	^49^
Case 2	**faeces, liver**	*Salmonella* spp. isolation	positive (*S*. 1,4,[5],12:i:-)	^38^
	**brain, lung, PSC ln, TB ln, liver, spleen, kidney, blood**	standard aerobic bacterial culture	positive (*S*. 1,4,[5],12:i:-)	—
	brain	*Listeria* spp. isolation	negative	^38^
	brain, lung, PS ln, TB ln, liver, spleen	*Brucella* spp. isolation	negative	^38^
	brain, lung, tonsils, PS ln, TB ln, liver, spleen, kidney, blood	*Brucella* spp. (PCR)	negative	^50^
	serum, HA, CSF	anti*- Brucella* spp. AB detection (S.A.R.)	negative	^52^
	**brain, lung, tonsils**, PS ln, **TB ln**, liver, **spleen, kidney, bladder**, blood	*Morbillivirus* (PCR, sequence analysis)	positive *(Dolphin Morbillivirus)*	^27^
	**brain**, lung, PSC ln, TB ln, spleen, kidney, bladder, pancreas	*Morbillivirus* (IHC)	positive	^25^
	brain, lung, TB ln, spleen, kidney, bladder	*Morbillivirus* (isolation)	negative	^51^
	serum, HA, CSF	anti*-Morbillivirus* AB detection (SN)	negative	^53^
	**brain**, PS ln, TB ln, liver, spleen, **heart**, muscle	*Toxoplasma gondii* (PCR)	positive	^26^
	**brain**, heart	*Toxoplasma gondii* (IHC)	positive	^25^
	**serum, HA**, CSF	*anti-T. gondii* AB detection (IFAT)	positive (serum,1:160; HA, 1:5120)	^25^
	brain, lung, **PSC ln**, TB ln, spleen kidney	*Herpesvirus* (PCR, sequence analysis)	positive *(Alphaherpesvirus)*	^49^
Case 3	**faeces**	*Salmonella* spp. isolation	positive (*S*. 1,4,[5],12:i:-)	^38^
	**brain, lung, PSC ln, PUL ln, liver, spleen, kidney**	*Salmonella* spp. isolation (with pre-enrichment)	positive (*S*. 1,4,[5],12:i:-)	^38^
	brain	*Listeria* spp. isolation	negative	^38^
	brain	*Brucella* spp. isolation	negative	^38^
	brain, lung, PSC ln, kidney	*Brucella* spp. (PCR)	negative	^50^
	serum, HA, CSF	anti*- Brucella* spp. AB detection (S.A.R.)	negative	^52^
	**brain, lung**, **PSC ln, PUL ln**, **liver, spleen, kidney, bladder**	*Morbillivirus* (PCR, sequence analysis)	positive *(Dolphin Morbillivirus)*	^27^
	brain, lung, PSC ln, PUL ln, liver, spleen, kidney, bladder	*Morbillivirus* (isolation)	negative	^51^
	serum, HA, CSF	anti*-Morbillivirus* AB detection (SN)	negative	^53^
	brain, PSC ln, PUL ln, liver, spleen, heart, muscle	*Toxoplasma gondii* (PCR)	negative	^26^
	serum, HA, CSF	*anti-T. gondii* AB detection (IFAT)	negative	^25^
	brain, lung, **PSC ln**, PUL ln, **liver**, spleen	*Herpesvirus* (PCR, sequence analysis)	positive *(Alphaherpesvirus)*	^49^

Legend - PSC ln: prescapular lymph node; TB ln: tracheo-bronchial lymph node; AB: antibodies; S.A.R: rapid serum agglutination; IHC: immunohistochemistry; IFAT: indirect immunofluorescence test; HA, humor acqueous; CSF: cerebrospinal fluid; PUL ln: pulmonary lymph node; SN: serum neutralization.

Tissues positive are shown in bold.

### Case 1

The first animal, a 204 cm (Total Length, TL) adult female, was found stranded in Ceriale (SV) (44.09N, 8.23E) on 2^nd^ February 2015, in a moderate nutritional status and a *post-mortem* condition code 3 (moderately decomposed). The estimated age was 17 years. Significant gross necropsy findings included a severe state of dehydration (related to prolonged exposure to dry and windy conditions before necropsy), a widespread internal congestion and a mild *Phyllobotrium delphini* and *Monorygma grimaldi* infection. The forestomach contained scanty material, represented by few highly digested cephalopod beaks, correlated to a non-recent meal. The cephalopod species were identified as 1 *Goliteuthis armata* (family Cranchiidae), typical of offshore and deep pelagic waters, 2 *Loligo vulgaris* (family Loginidae), which generally inhabits coastal waters and 6 *Todarodes sagitattus* (family Ommastrephidea), a species widely distributed both in coastal and offshore waters.

Microscopically, a non-suppurative meningoencephalitis characterized by vasculitis, gliosis, meningitis and prominent perivascular mononuclear cell cuffing was observed in all brain areas, associated with one protozoan cyst located in the frontal cortex.

Despite the poor *post-mortem* preservation degree, a mild broncho-interstitial pneumonia with *oedema* and multifocal parasitic granulomas, multifocal chronic cholangiohepatitis, lymphoid depletion and a mild mixed inflammatory infiltrate in spleen, were observed.

The diagnosis of *Salmonella* infection was achieved by the isolation of *Salmonella* 1,4,[5],12:i:- from faeces (intestine) and liver using a specific bacteriological approach based on selective broth enrichment and subculture onto selective solid media, as shown in Table 2.

**Table 2:** *Salmonella* spp. isolation from the 3 striped dolphins (*Stenella coreuleoalba*)under study: methods, results and classification of *Salmonella* infection.

	Method	Tissues	Infection
Case 1	A	brain-lung-prescapular and tracheobronchial lymph nodes-liver-spleen-kidney	sub-clinic/asymptomatic
	B	**feces (intestine)-liver**	
Case 2	A	**brain-lung-prescapular and tracheobronchial lymph nodes**-**live**r-**spleen**-**kidney-blood**	septicaemic
	B	**feces (intestine)-liver**	
Case 3	B	**feces (intestine)**	disseminated
	C	**brain-lung-prescapular and pulmonary lymph nodes-liver-spleen-kidney**	

Legend –

A: standard aerobic bacterial culture;

B: enrichment step in selective liquid media (Selenyte Cystine broth-SC and Mueller-Kaufmann Tetrathionate-Novobiocin –MKTTn broth) and subculture onto selective solid media (Brilliant Green Agar –BGA and Xylose Lysine Desoxycholate –XLD agar);

C: pre-enrichment step in Buffered Peptone Water (BPW) and subculture onto semisolid medium (Rappaport Vassiliadis Semi-Solid Medium Modified-MSRV agar).

Tissues positive are shown in bold.

The antimicrobial susceptibility results are reported in Table 3.

**Table 3:** Antimicrobial phenotipic profiles of the 3 *Salmonella* 1,4,[5],12:i:- isolates recovered from the 3 striped dolphins under study (R = resistant; I = intermediate; S = susceptible).

	A	AMC	C	CAZ	CIP	CTX	G	K	KF	NAL	S	SXT	T
Case 1	R	I	S	I	S	I	S	S	I	I	R	I	R
Case 2	R	I	S	S	S	S	S	S	S	S	R	R	R
Case 3	R	S	S	S	S	S	S	S	S	S	R	S	R

Abbreviations: A, ampicillin; AMC, amoxicillin/clavulanic acid; C, chloramphenicol; CZ, ceftazidime; CIP, ciprofloxacin; CTX, cefotaxime; G, gentamicin; K, kanamycin; KF, cefalotin; NAL, nalidixic acid; S, streptomycin; SXT, trimethoprim-sulfamethoxazole; T, tetracycline.

The Multiple-Locus Variable-number tandem-repeat Analysis (MLVA) profile associated to this isolate was 3-13-12-n.a.-0211 (Table 4).

**Table 4:** Biomolecular typing results related to MLVA, *in silico* MLST and AMR genotype.

	MLVA	MLST	AMR genotype	Template	Related Phenotype	query_coverage
Case 1	3-13-12-n.a.-0211	ST34	ASSuT	aph(3″)-Ib_5_AF321551	Streptomycin-Resistance	100.00
				aph(6)-Id_1_M28829	Aminoglycoside Resistance	100.00
				blaTEM-1B_1_JF910132	Beta-lactam Resistance	100.00
				sul2_3_HQ840942	Sulphonamide Resistance	100.00
				tet(B)_4_AF326777	Tetracycline Resistance	100.00
Case 2	3-16-8-n.a.-0211	ST34	ASSuT	aph(3″)-Ib_5_AF321551	Streptomycin-Resistance	100.00
				aph(6)-Id_1_M28829	Aminoglycoside Resistance	100.00
				blaTEM-1B_1_JF910132	Beta-lactam Resistance	100.00
				sul2_3_HQ840942	Sulphonamide Resistance	100.00
				tet(B)_4_AF326777	Tetracycline Resistance	100.00
Case 3	3-15-8-n.a.-0211	ST34	ASSuT	aph(3″)-Ib_5_AF321551	Streptomycin-Resistance	100.00
				aph(6)-Id_1_M28829	Aminoglycoside Resistance	100.00
				blaTEM-1B_1_JF910132	Beta-lactam Resistance	100.00
				sul2_3_HQ840942	Sulphonamide Resistance	100.00
				tet(B)_4_AF326777	Tetracycline Resistance	100.00

Abbreviations: MLVA, Multiple-Locus Variable-number tandem-repeat Analysis; MLST, Multilocus Sequence Typing; AMR, Antimicrobial Resistance; ST, Sequence Type; ASSuT, Ampicillin, Streptomycin, Sulfonamide and Tetracycline resistant isolate.

The multilocus sequence typing (MLST) *in silico* showed that *Salmonella* 1,4,[5],12:i:- isolate belonged to ST34 (Table 4); the results of KmerResistance 2.2 are listed in Table 4 and the results of SPIFinder 1.0 “Pathogenic Island” are listed in Table 5.

**Table 5:** Salmonella Pathogenicity Islands extracted using SPIFinder 1.0.

	Gene	Origin	%Identity	HSP/Query length	Insertion location	SPI Accession
Case 1	SPI-1	Salmonella Typhimurium LT2	100.00	44279/44279	fhlA-mutS	gi|16763390:3005842-3050120
	SPI-2	Salmonella Typhimurium LT2	100.00	40071/40071	tRNA-valV	gi|16763390:1461740-1501810
	SPI-3	Salmonella Typhimurium LT2	99.99	16616/16616	tRNA-selC	gi|16763390:3948576-3965191
	SPI-4	Salmonella Choleraesuis str SC-B67	98.94	26699/26698	ssb-soxSR	gi|62178570:4411902-4438599
	SPI-5	Salmonella Typhimurium LT2	100.00	9069/9069	tRNA-serT	gi|16763390:1175321-1184389
	SPI-13	Salmonella Gallinarum SGD-3	99.41	338/338	tRNA-pheV	AY956832
	SPI-13	Salmonella Gallinarum SGG-1	100.00	404/404	tRNA-pheV	AY956833
	SPI-13	Salmonella Gallinarum SGA-10	100.00	341/341	tRNA-pheV	AY956834
	SPI-14	Salmonella Gallinarum SGA-8	100.00	501/501	Not_published	AY956835
	SPI-14	Salmonella Gallinarum SGC-8	99.55	441/441	Not_published	AY956836
	C63PI	Salmonella Typhimurium SL1344	99.88	21435/25252	fhlA	AF128999
Case 2	SPI-1	Salmonella Typhimurium LT2	100.00	44280/44279	fhlA-mutS	gi|16763390:3005842-3050120
	SPI-2	Salmonella Typhimurium LT2	100.00	40071/40071	tRNA-valV	gi|16763390:1461740-1501810
	SPI-3	Salmonella Typhimurium LT2	99.99	16616/16616	tRNA-selC	gi|16763390:3948576-3965191
	SPI-4	Salmonella Choleraesuis str SC-B67	98.94	26699/26698	ssb-soxSR	gi|62178570:4411902-4438599
	SPI-5	Salmonella Typhimurium LT2	100.00	9069/9069	tRNA-serT	gi|16763390:1175321-1184389
	SPI-13	Salmonella Gallinarum SGG-1	100.00	404/404	tRNA-pheV	AY956833
	SPI-13	Salmonella Gallinarum SGA-10	100.00	341/341	tRNA-pheV	AY956834
	SPI-13	Salmonella Gallinarum SGD-3	99.41	338/338	tRNA-pheV	AY956832
	SPI-14	Salmonella Gallinarum SGC-8	99.55	441/441	Not_published	AY956836
	SPI-14	Salmonella Gallinarum SGA-8	100.00	501/501	Not_published	AY956835
	C63PI	Salmonella Typhimurium SL1344	99.91	21140/25252	fhlA	AF128999
Case 3	SPI-1	Salmonella Typhimurium LT2	100.00	44280/44279	fhlA-mutS	gi|16763390:3005842-3050120
	SPI-2	Salmonella Typhimurium LT2	100.00	40071/40071	tRNA-valV	gi|16763390:1461740-1501810
	SPI-3	Salmonella Typhimurium LT2	99.99	16616/16616	tRNA-selC	gi|16763390:3948576-3965191
	SPI-5	Salmonella Typhimurium LT2	100.00	9069/9069	tRNA-serT	gi|16763390:1175321-1184389
	SPI-13	Salmonella Gallinarum SGG-1	100.00	404/404	tRNA-pheV	AY956833
	SPI-13	Salmonella Gallinarum SGA-10	100.00	341/341	tRNA-pheV	AY956834
	SPI-14	Salmonella Gallinarum SGA-8	100.00	501/501	Not_published	AY956835
	SPI-14	Salmonella Gallinarum SGC-8	99.55	441/441	Not_published	AY956836
	C63PI	Salmonella Typhimurium SL1344	99.91	25244/25252	fhlA	AF128999
	SPI-13	Salmonella Gallinarum SGD-3	99.41	338/338	tRNA-pheV	AY956832

Abbreviations: SPI, Salmonella Pathogenic Island.

*Aeromonas hydrophila* was additionally isolated from brain, spleen and lung by aerobic bacterial culture, while neither *Listeria* spp nor *Brucella* spp. were isolated.

No biomolecular evidence of *Brucella* spp. was found, with no anti-*Brucella* spp. antibodies being detected in the blood serum.

*Dolphin Morbillivirus* (DMV) infection targeting the lung and prescapular lymph node was demonstrated by means of PCR, followed by amplicon sequencing and BLAST analysis. Nevertheless, viral isolation attempts were negative, with no anti-*Morbillivirus* antibodies being detected in serum. Furthermore, no immunohistochemical (IHC) evidence of morbilliviral antigens was found in tissues from this dolphin.

Direct evidence of *Toxoplasma gondii* infection was obtained through the observation of one protozoan cyst, coupled with PCR positivity in the animal’s brain, alongside simultaneous evidence of anti-*T. gondii* antibodies in serum (>1:160). No IHC evidence of *T. gondii*-specific antigens was additionally found in tissues from this dolphin.

No biomolecular evidence of *Herpesvirus* was found in the examined tissues.

### Case 2

The second animal, a 202 cm (TL), adult female, was found stranded dead in Savona (44.29N, 8.45E) on 14^th^ September 2017, in a poor nutritional status and in *post-mortem* condition code 2 (fresh). The estimated age was 15 years. This was a lactating individual. Relevant pathological findings included haemorrhagic suffusions at blubber and myocardal levels, along with a severe *Phyllobotrium delphini* and *Monorygma grimaldi* infection, a mild pulmonary congestion, a multicentric reactive lymphadenopathy associated with splenic hypertrophy and the presence of numerous, focal ulcers in the keratinized gastric compartment. The forestomach contained scanty ingesta, mostly represented by undigested seagrass (*Posidonia oceanica*).

**Figure 3:**
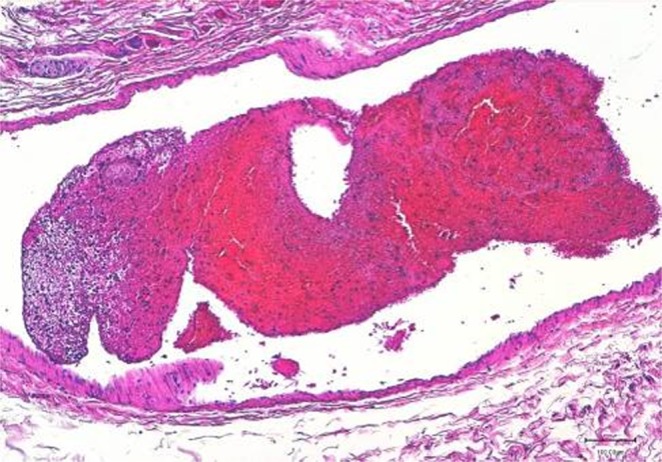
Striped dolphin (*Stenella coeruleoalba*). Intestinal mesentery. A large embolus is clearly shown inside the *lumen* of a blood vessel. 20x HE (created by K.V.).

Microscopically, a non-suppurative meningoencephalitis characterized by multifocal malacic areas associated with *oedema*, gitter cells, mononuclear cell infiltrates, perivascular cuffs, syncytial cells and multiple protozoan cysts, some of which destroyed, were observed. Areas characterized by gliosis and neuronophagia, together with focal and moderate non-suppurative meningitis, were also apparent. One protozoan cyst, characterized by a huge diameter, without reaction in the surrounding tissue, was evident at parietal cortex level. Protozoan cysts were also detected in the heart. A severe membranous glomerular nephropathy, associated with multifocal interstitial chronic nephritis, was additionally found. Lymphoid depletion and sinus hypercellularity were observed in spleen and lymph nodes. Mild endocardiosis of the left atrio-ventricular valve, with fibrosis and lymphocytic epicarditis, were also found. Multifocal superficial erosions of the forestomach mucosa and necrosis of the intestinal crypts’ epithelium were observed. An intravascular embolus in the intestinal mesentery was also detected (Fig. 3).

The diagnosis of a septicaemic form of salmonellosis was achieved by the isolation of *Salmonella* 1,4,[5],12:i:- from samples of brain, lung, prescapular and tracheo-bronchial lymph nodes as well as from liver, spleen, kidney and blood, by standard aerobic bacterial culture, and from feces (intestine) and liver using a a specific bacteriological approach based on selective broth enrichment and subculture onto selective solid media (Table 2).

The antimicrobial susceptibility results, corresponding to a multidrug resistant phenotype (ASSuT isolate, Ampicillin, Streptomycin, Sulphonamide and Tetracycline resistant), are reported in Table 3.

The MLVA profile associated to this isolate was 3-16-8-n.a.-0211 (Table 4).

*In silico* MLST showed that *Salmonella* 1,4,[5],12:i:- isolate belonged to ST34 (Table 4); the results of KmerResistance 2.2 are listed in Table 4 and the results of SPIFinder 1.0 “Pathogenic Island” are listed in Table 5.

No other significant bacteria were isolated, including *Listeria* spp. and *Brucella* spp.

No biomolecular evidence of *Brucella* spp. was found, with no anti-*Brucella* spp. antibodies were being detected in serum, humor acqueous or cerebrospinal fluid.

A generalized DMV infection was demonstrated, by means of PCR, in brain, lung, laryngeal tonsils, tracheo-bronchial lymph node, spleen, kidney and bladder, being subsequently confirmed through amplicon sequencing and BLAST analysis. *Morbillivirus*-specific antigens were additionally detected in brain by IHC. Nevertheless, no anti-*Morbillivirus* antibodies were detected in serum, humor aqueous or cerebrospinal fluid and virus isolation attempts failed.

Direct evidence of *T. gondii* infection was obtained through the observation of protozoal cysts and PCR positivity in brain and heart, together with the simultaneous occurrence of anti -*T. gondii* antibodies in serum (1:160) as well as in humor aqueous (1:5120), IHC evidence of *T. gondii* - specific antigens was also found in brain.

Furthermore, PCR investigations, followed by amplicon sequencing and BLAST analysis, yelded positive results for cetacean *Alphaherpesvirus* at the level of prescapular lymph node.

Among the different organochlorine (OC) tissue concentrations, hexachlorobenzene (HCB) was the compound with the lowest levels (47.94 ng/g l.w.) followed by dichlorodiphenyltrichloroethane and its metabolites (DDTs) (10074.67 ng/g l.w.) and polychlorinated biphenyls (PCBs) (41059.22 ng/g l.w.). The Extracted Organic Material percent (EOM%) was 50.66, showing a relevant depletion of blubber layer. The PCB levels were greater than the estimated toxicity threshold (17 mg/kg l.w.)^21,22^, confirming that the specimen experienced deleterious health effects.

### Case 3

The third animal, a 204 cm (TL), adult male, was found stranded dead in Vado (SV) (44.26N, 8.45E) on 20^th^ January 2018, in good nutritional status and in *post-mortem* condition code 4 (decomposed). The estimated age was 18 years. Significant gross necropsy findings included a generalized *post-mortem* autolysis, with loss of more than 50% of the skin, associated with a mild *Phyllobotrium delphini* and *Monorygma grimaldi* infection and 2 *Pholeter gastrophilus* gastric nodules.

The forestomach contained scanty material, represented by few fully digested cephalopod beaks, correlated to a non-recent meal. The cephalopod species were identified as 1 *Ancitroteuthis lichtensteini* (family Onychiteuthidae) and 3 *Todarodes sagittatus* (family Ommastrephidae), both of which are normally found in coastal and pelagic waters.

Considering the advanced stage of *post-mortem* autolysis, a limited panel of investigations was performed and histology and immunohistochemistry were not attempted.

The diagnosis of a generalized form of salmonellosis was achieved by the isolation of *Salmonella* 1,4,[5],12:i:- initially just from faeces (intestine), using a specific bacteriological approach based on selective broth enrichment and subculture onto selective solid media (Table 2). This positive finding subsequently led us to carry out supplementary investigations, in order to ascertain the potential dissemination of the infection. Considering the features of the samples available, represented by brain, lung, prescapular and pulmonary lymph nodes, liver, spleen and kidney, to enhance the diagnostic sensitivity the isolation was therefore attempted using a specific isolation protocol, based on non selective pre-enrichment broth and subculturing on semi-solid media, with positive results in all tissues tested (Table 2).

The antimicrobial susceptibility results are reported in Table 3.

The MLVA profile associated to this isolate was 3-15-8-n.a.-0211 (Table 4).

*In silico* MLST showed that *Salmonella* 1,4,[5],12:i:- isolate belonged to ST34 (Table 4); the results of KmerResistance 2.2 are listed in Table 4 and the results of SPIFinder 1.0 “Pathogenic Island” are listed in Table 5.

Neither *Listeria* spp. nor *Brucella* spp. were isolated from brain.

No biomolecular evidence of *Brucella* spp. was found nor anti-*Brucella* spp. antibodies were detected.

Furthermore, biomolecular evidence of systemic DMV infection was obtained through PCR positivity in brain, lung, prescapular and pulmonary lymph nodes, liver, spleen, kidney and bladder, being subsequently and confirmed by amplicon sequencing and BLAST analysis. Nevertheless, no anti-*Morbillivirus* antibodies were detected in serum, humor aqueous or cerebrospinal fluid and virus isolation attempts were all negative.

No microscopic nor IHC and biomolecular (PCR) evidence of *T. gondii* infection was found, with no anti-*T. gondii* antibodies being additionally detected in this dolphin’s blood serum, humor aqueous and cerebrospinal fluid.

Finally, PCR investigations, followed by amplicon sequencing and BLAST analysis, yelded positive results for cetacean *Alphaherpesvirus* in the liver and prescapular lymph node.

The tissue concentrations of HCB, DDTs and PCBs in blubber were 173.84 ng/g l.w., 19809.83 ng/g l.w. and 35757.92 ng/g l.w. respectively, and also in this case the PCB levels were greater than the toxicity threshold of 17 mg/kg l.w^21,22^. The EOM% was very high (94.95). In this specimen the toxicological stress was evaluated also using a theoretical model^23^ using DDT and PCB levels expressed in ng g-1 dry weight (d.w.): the result of Canonical Variable (CAN) was −0.514, so it did not reveal the presence of hazardous levels of OC pollutants (CAN > 0.47).

## Discussion

The herein presented results clearly show that cetaceans in the Pelagos Sanctuary deal with some pathogens of anthropogenic origin (e.g. *Salmonella* spp., *Toxoplasma gondii*), which may adversely affect both their individual health and their population conservation *status*.

Moreover, the interaction of multiple pathogens, as represented in these cases, may have significantly impacted the course and severity of the disease conditions experienced by the herein investigated dolphins, leading to their subsequent stranding and death.

Considering the infection by a monophasic variant of *Salmonella* Typhimurium, the first case (Case 1) was characterized by the presence of the bacterium only on faeces and liver, the latter possibly related to bacterial migration from the intestine to the liver in a moderately decomposed carcass (Code 3). In this case, the striped dolphin was supposed to carry *Salmonella* asymptomatically, thus harbouring the bacterium in faeces and shedding into the surrounding environment without displaying clinical symptoms.

We hypothesize a multifactorial cause of death for this individual, involving a chronic systemic DMV infection, confirmed by molecular analysis from lung and prescapular lymph node and specific lesions in spleen, which could have seriously compromised the health status, along with an additional impairment likely caused by cerebral toxoplasmosis, which may have led in turn to the animal’s disorientation and subsequent stranding.

The second case (Case 2) was instead characterized by the isolation of *Salmonella* 1,4,[5],12:i:- from multiple tissues and/or biological fluids, including blood samples. *Salmonella* was the only primary bacterial pathogen detected, in a fresh carcass (Code 2), as the cause of a septicaemic form of infection associated with microscopic lesions (intestinal necrosis, vascular embolus in intestinal mesentery). Such severe evolution of the infection likely played a role in this animal’s stranding and death and it could be related to a multifactorial impairment of this individual. Herein, we hypothesize that a subacute systemic *Morbillivirus* infection, associated with *Herpesvirus* infection and hazardous levels of pollutants, could have impacted the severity of toxoplasmosis, and that the cerebral impairment may have led to the animal’s disorientation and behavioural changes, such as debris ingestion^24^. The role of the ulcers in stomach, possibly related to gastric pain following the ingestion of abnormal material as a substitute of fish^25–27^, must not be underestimated, as they could have acted as a door of entry for *Salmonella* infection, due to the ingestion of abnormal, contaminated material (*Posidonia oceanica*, found in coastal polluted waters) in a weakened individual, incapable of catching.

In the third case (Case 3), a diagnosis of a disseminated form of salmonellosis was obtained thanks to supplementary investigations performed after the first isolation of *Salmonella* from faeces (intestine). The isolation from multiple tissues like brain, lung, prescapular and pulmonary lymph nodes, spleen and kidney, in addition to the liver, cannot be related to a *post-mortem* migration of bacteria from the intestine to contiguous and/or more distant organs; therefore, considering the lack of information for the few ancillary tests available, a septicaemic form of infection, with a likely high rate of bacterial dissemination, cannot be ruled out. The systemic DMV infection, confirmed by molecular analysis from multiple tissues, including blood, associated with *Alphaherpesvirus* infection, could have debilitated and predisposed the animal to a fatal systemic infection by *Salmonella* 1,4,[5],12:i:-.

Considering the data retrieved from molecular characterization, the *Salmonella* 1,4,[5],12:i:- isolates detected in the 3 herein investigated dolphins appear to be highly related to each other. In particular, all the isolates, as shown by KmerResistance 2.2 tool (Table 4), harboured the same genes for resistance to ampicillin, streptomycin, sulphonamides, tetracycline and are identifiable as ASSut, which is described as one of the most common profiles of resistance circulating in Italy and in Europe associated to monophasic variant of *Salmonella* Typhimurium^28^. Case 1 and Case 2 share the same Salmonella Pathogenic Island (SPI) genes’ profiles, with Case 3 differing only for the absence of SPI-4 (Table 5).

Furthermore, due to the results of *in silico* molecular typing, all isolates belonging to the same MLST type (Sequence Type, ST) and the MLVA results show a high correlation (Table 4).

Based on the query performed on the MLVA database profiles of Italian Reference Laboratory for Salmonellosis, the MLVA profile related to Case 1 (3-13-12-n.a.-0211) was found to be associated with 38 *Salmonella* isolated namely of human (N = 15), swine (N = 22) and shellfish (N = 1) origin, respectively (Table 6). All profiles were epidemiologically unrelated to each other.

**Table 6:** MLVA profile associated to the cases described in this work, compared to database MLVA of Italian Reference Laboratory for Salmonellosis.

	MLVA Profile Case 1 3-13-12-n.a.-0211 (no of isolates)	MLVA Profile Case 2 3-16-8-na-0211 (no of isolates)	MLVA Profile Case 3 3-15-8-na-0211 (no of isolates)
Sources	human (N = 15)	swine (N = 1)	swine (N = 3)
	swine (N = 22)		poultry (N = 1)
	shellfish (N = 1)		cat (N = 1)

Legend – MLVA: Multiple Locus Variable number tandem repeats Analysis.

These findings suggest that such specific MLVA profile is quite common in swine and human sources; however, they also highlight the need to deeply investigate the role of seawater environment in the spread of this zoonotic pathogen.

The MLVA profiles concerning Cases 2 and 3 (3-16-8-n.a.-0211 and 3-15-8-n.a.-0211, respectively) are tightly related, differing by a unique repeat unit occurring on STTR5 locus (Short Typhimurium Tandem repeat).

The MLVA profile of Case 2 was not already present into the database, differently from that of Case 3, which was found to be associated with 5 isolated of swine (N = 3), poultry(N = 1) and cat (N = 1) origin, respectively, overall collected from 2017 to 2018 (Table 6).

Moreover, Single Nucleotide Polymorphisms (SNP) analysis confirmed a high degree of correlation between Cases 2 and 3, as shown in the SNP matrix reported in Table 7, with only 2 different SNPs; when Cases 2 and 3 were compared with Case 1, they both differed from Case 1 by 38 SNPs. The genetic differences between these isolates may indicate that Cases 2 and 3 belong to the same clonal cluster, while Case 1 can be placed on the threshold, identifying a different clonal cluster, even if a threshold SNP number for *Salmonella* might not be identifiable^29^ and, to our knowledge, has not been defined yet for *Salmonella* 1,4,[5],12:i:-.

**Table 7:** SNP matrix inferred by the mean of CSI Phylogeny 1.4 (https://cge.cbs.dtu.dk/services/CSIPhylogeny/) to represent the phylogenetic relationships between strains.

	Case 1	Case 2	Case 3
Case 1	0	38	38
Case 2	38	0	2
Case 3	38	2	0
min: 2 max: 38			

Abbreviations: SNP, Single Nucleotide Polymorphism.

Although the source of infection by this serovar to the dolphins remains unknown, different considerations and hypotheses can be drawn about the *Salmonella* contamination of Pelagos Sanctuary marine waters and the transmission pathways for these striped dolphins.

*Salmonella* spp. is a marker for faecal contamination. The waters of highly populated coastal areas, like those of the Pelagos Sanctuary, receive large quantities of wastewater discharged from human, animal and industrial sources, treated and sometimes untreated.

Based on available data of the Regional Reference Laboratory for Salmonella typing of the Istituto Zooprofilattico (CeRTIS), retrospectively investigated, the circulation of *S*.1,4,[5],12:i:- at human, animal and environmental levels has increased in recent years in the Liguria Region as well as in whole Italy. Indeed, this serovar was isolated from 32 patients suffering from salmonellosis in all provinces of Liguria, in 2016, 2017 and January 2018, as well as from 6 wild boars sampled in the Genoa province, in 2014 and 2015, and from one environmental sample, in the Genoa province, at the beginning of February 2018. No positivity was found for for *Salmonella* spp. in seafood (fish, mussels, edible sea molluscs) submitted to official controls carried out on the coastline from 2015.

In the area surrounding the Ligurian coastal zone there is not a significant livestock production, but a consistent presence of pig farms characterizes Corsica as well as Tuscany and Northern Sardinia, the coasts of which are encompassed in the Pelagos Sanctuary. Therefore, along with human sewage, untreated wastewater discharged from pig farms might also represent a potential source of *Salmonella* in the concerned area.

In addition, considering the significant presence of wild boars in Liguria, Tuscany and Corsica, the risk of bacterial shedding through their faeces should not be underestimated.

Moreover, rivers, rainfall and extreme weather events may transfer enteric pathogens from distant sources to coastal waters^8,30^. Noteworthy, the contamination of Pelagos Sanctuary marine waters by bacteria originating from human and/or animal wastes could have been potentially increased by the severe flooding repeatedly occurred on the Ligurian and Tuscany coasts in recent years (Genova 2014, Nice 2016, Livorno 2017).

As the Mediterranean basin represents one of the busiest navigation crossroads and top tourist destinations in the world, an additional potential source of bacterial contamination in the Pelagos Sanctuary could be represented by wastewaters discharged from ships and boats^31^. The presence of marine birds represents an additional potential reservoir of infection, and in this regard the last tract of the Magra River, in Liguria, characterized by watersheds highly populated by water birds, should be also taken into account as a potential contamination source.

Additionally, focusing on the eating habits of *Stenella coeruleoalba*, the transmission of *Salmonella* infection could be derived from the consumption of infected or contaminated marine organisms like cephalopods or filter-feeders clupeids.

Furthermore, keeping in mind the typical respiratory patterns in cetaceans, the aerolized sea surface microlayer (SML), laden with microorganisms and contaminants, could have been inhaled deep into the tracheobronchial tree during porpoising, being subsequently deposited into alveolar spaces with more forceful exhalation and inhalation processes^32^.

Analysing more in depth the features of the striped dolphin stranded in 2017 (Case 2), another hypothesis could be represented by direct ingestion of contaminated water while trying to eat something or by assumption of contaminated seagrass, which does not represent a common food source for any cetacean.

The potential impact that *Salmonella* could have on offshore, resident, or transient populations is unknown and deserves further considerations. In order to better define the role that *Salmonella* plays in causing disease in cetaceans it would be appropriate to test all marine mammals found stranded for this pathogen and perform antimicrobial sensitivity tests on all *Salmonella* isolates. The detection of multiresistant isolates, as shown in these cases, has important implications, confirming the seepage of land-based antimicrobial compounds and/or antibiotic resistant microorganisms into the marine environment, coupled with the bioaccumulation, and possible biomagnification, into the highest marine trophic levels^32,33^.

The long survival of *Salmonella* spp in seawater, up to 17 months^9^, supports even more the health risks for susceptible marine animals and humans.

In conclusion, these results highlight the role of cetaceans as sentinel species for zoonotic and terrestrial pathogens in the marine environment and suggest a high level of seawater contamination in the North Western Italian coast, which may adversely impact both cetacean species and public health.

Moreover, the herein reported findings represent an additional, relevant matter of concern for the zoonotic importance of the *Salmonella* serovar isolated from the dolphins under investigation, which is considered as the emerging one in humans in Europe^34^.

The potential risk of *Salmonella* isolates circulating in the marine environment to public health should not be discounted^9^, considering the bacterial release from both carriers and infected marine mammals, along with the spread of multiresistant isolates and the medical implications for humans sharing the same habitat or working with marine mammals^32^.

Our observations indicate that *Salmonella* 1,4,[5],12:i:- is characterized by a potential pathogenic role in striped dolphins and, consequently, it should be added to the list of pathogenic bacteria causing generalized infections in cetaceans.

Moreover, these results suggest cetaceans as novel potential *reservoir* for one of most important *Salmonella* serovars.

In order to characterize the possible transmission routes, further research is needed to determine if this zoonotic pathogen poses a significant risk to cetaceans, by investigations on the infections’s prevalence as well as on its pathogenicity and epidemiology and the comparison of isolates from wild birds, wild mammals, livestock, agricultural sources and clinical samples, using not only Pulsed-field gel electrophoresis (PFGE) and MLVA^35^ but also Whole Genome Sequencing typing (WGS). Specific investigations for *Salmonella* spp. on surface river and coastal waters would be also an appropriate tool to assess the levels of contamination in the aquatic environments under consideration, while the reduction of run-offs, erosion and urban pollution in coastal areas, by an *ad hoc* surveillance on public waterways, would be an appropriate additional measure, preventing marine environmental contamination by *Salmonella* spp. as well as by other oro-faecally transmitted microorganisms.

## Materials and Methods

### Materials

The carcasses of three striped dolphins, stranded along the Ligurian Sea coast between 2015 and the beginning of 2018 (January-March), were submitted for necropsy to Istituto Zooprofilattico Sperimentale, Diagnostic Laboratory of Imperia, where a complete examination was performed according to standard protocols^36^.

The animals’ age was estimated by counting dentine growth layers (GLGs) on longitudinal section of teeth^37^. Three mandibular teeth collected from each specimen were mounted in epoxy resin and cut along the sagittal plane in several sections (0,3 mm thick), using a Low Spread Saw endowed with a diamond blade. The total number of GLGs in each of the tooth sections was determined in three separate sessions by three independent readers.

Gastric contents were collected during necropsies and preserved frozen for later analysis. They were then weighed, filtered and sorted, and the species were identified to the lowest taxonomic level possible. The foreign bodies were also weighed and sorted.

During necropsy, the tissue samples of all the major organs and lesions were collected and split into aliquots for subsequent analyses: one was kept frozen at −20 °C for microbiological and toxicological investigations, one at −80 °C for biomolecular and virological analyses, and the other was preserved in 10% buffered formalin for histological and immunohistochemical (IHC) investigations. Blood serum, aqueous humour and cerebrospinal fluid (CFS) were collected, when available, and kept frozen at −20 °C for serological investigations.

### Microbiology

Tissue samples including brain, lung, prescapular and tracheobronchial lymph nodes, liver, spleen and kidney (Cases 1 and 2) and blood (Case 2) were processed for standard aerobic, anaerobic and microaerobic (5% CO_2_) bacterial culture and identification, by biochemical and/or molecular analyses. Following international recommendations^38^, samples from target tissues underwent specific bacteriological procedures to screen *Listeria* spp. and *Brucella* spp. (Case 1 and 2). Samples of brain (Case 3), recovered retrospectively, underwent the same specific bacteriological approach.

### *Salmonella* isolation, identification and typing

Samples of liver and faeces (intestine) (Case 1 and 2), and of faeces (intestine) (Case 3) underwent specific bacteriological procedures to screen *Salmonella* spp. by means of an enrichment step in selective liquid media (Selenyte Cystine broth, Microbiol, Cagliari; MKTTn broth, Liofilchem, Roseto degli Abruzzi, TE) for 24 h at 37 °C, followed by subculture on solid media (BGA and XLD agar, Oxoid, Rodano, MI) for 24 hr at 37 °C^38^.

Samples of brain, lung, prescapular and pulmonary lymph nodes, liver, spleen and kidney (Case 3), recovered retrospectively, underwent specific bacteriological procedures for *Salmonella* spp. in order to enhance the diagnostic sensitivity, by means of a pre-enrichment step in Buffered Peptone Water (BPW, Oxoid, Rodano, MI), followed by subculture on semisolid medium (MSRV agar, Oxoid, Rodano, MI) for 48 hrs at 42 °C^38^.

*Salmonella* spp. were identified based on colony morphology on selective media and biochemical properties, according to ISO 6579-1:2017^39^.

Serotyping was carried out according to the Kauffman-White scheme^40^.

### Salmonella antimicrobial susceptibility testing

The Kirby–Bauer disc diffusion test was performed using Mueller-Hinton agar plates (Microbiol, Uta (CA), Italy) using the following antimicrobials and concentrations (μg):

ampicillin (A, 10), amoxicillin/clavulanic acid 2:1 (AMC, 30), chloramphenicol (C, 30), ceftazidime (CAZ, 10), cyprofloxacin (CIP, 5), cefotaxime (CTX, 5), gentamicin (G, 10), kanamycin (K, 30), cefalotin (KF, 30), nalidixic acid (NAL, 30), streptomycin (S, 10), trimethoprim–sulfamethoxazole (SXT, 1.25/23.75), and tetracycline (T, 30). Data were interpreted using CLSI guidelines (M100-S25, 2015).

Each bacterial isolate was classified as susceptible, intermediate, or resistant, depending on the growth inhibition diameter.

### *Salmonella* sequencing and Whole Genome Sequencing (WGS) typing

WGS of the DNA extracted from the *Salmonella* 1,4,[5],12:i:- isolates was performed on the MiSeq platform (Illumina, San Diego, United States) using paired-end libraries which were prepared by following the Nextera™ DNA Flex Library Prep Kit (Illumina, San Diego, United States), with 150-bp read length. The reads were first subjected to the Galaxy tool “FastQC Read Quality reports”, accessed via the Galaxy public server at https://usegalaxy.org ^41^, to provide the quality control checks on raw sequence data and then the reads were trimmed using the Galaxy tool Trimmomatic^42^. We used Unicycler (ver. 0.4.1.1) via Galaxy to assemble genomes^43^. The assembled genomes were processed to determinate the multilocus sequence typing (MLST) *in silico* with MLST 1.8 (accessed via https://cge.cbs.dtu.dk/services/MLST)^44^, to identify antimicrobial resistance genes with KmerResistance 2.2 (accessed via https://cge.cbs.dtu.dk/services/KmerResistance/)^45^, thereafter being investigated for Salmonella Pathogenicity Islands with SPIFinder 1.0 (accessed via https://cge.cbs.dtu.dk/services/SPIFinder/). The fastq files of paired-nd reads were processed with CSI Phylogeny 1.4 (accessed via https://cge.cbs.dtu.dk/services/CSIPhylogeny/) to call and filter single nucleotide polymorphisms (SNPs) and infer phylogeny based on the concatenated alignment of the high quality SNPs^46^.

Multiple Locus Variable number tandem repeat Analysis (MLVA) was performed as previously described^47^. MLVA results were reported as a string of five numbers (STTR9–STTR5–STTR6–STTR10pl–STTR3) (STTR: Short Typhimurium Tandem Repeat), representing the Variable Number of Tandem Repeats (VNTR) at the corresponding locus, or as n.a. in cases where a PCR product was not obtained for a locus. The MLVA nomenclature suggested by Larsson *et al*.^48^ was adopted in this study. The MLVA profiles obtained were compared with MLVA database profiles of Italian Reference Laboratory for Salmonellosis (over a period from 2015 to 2018).

### Histology and immunohistochemistry

Representative tissue samples from Case 1 (brain, lung, heart, liver, spleen, kidney, skeletal muscle, adrenal gland and reproductive system) and from Case 2 (brain, tonsils, lung, prescapular and tracheobronchial lymph nodes, heart, liver, spleen, pancreas, stomach, intestine, skeletal muscle, skin, kidney, urinary bladder, adrenal gland and reproductive system) were collected and fixed in 10% neutral buffered formalin, embedded in paraffin, sectioned at 4 ± 2 µm, stained with haematoxylin and eosin (H&E) and examined under a light microscope.

Five different areas from the brain were sampled, including the telencephalon, diencephalon, mesencephalon, cerebellum and brainstem (Cases 1 and 2).

Immunohistochemistry (IHC) for *Morbillivirus* was performed on tissue sections from Case 1 (brain, lung, spleen and kidney) and from Case 2 (brain, lung, prescapular and tracheo-bronchial lymph nodes, spleen, kidney, bladder and pancreas), using a monoclonal anti-*Canine Distemper Virus* (CDV) antibody (VMRD)^25^.

*Toxoplasma gondii* IHC was carried out on samples from Cases 1 and 2 (the five aforementioned brain areas, along with the H&E-stained sections of all tissues in which coccidian parasites were identified by histopathology and/or biomolecular tests)^25^, using a polyclonal serum of caprine origin (VMRD).

Considering the advanced *post-mortem* autolysis, tissue samples from Case 3 were not investigated.

### PCR, sequence analysis, virological investigations and serology

Molecular detection of *Dolphin Morbillivirus* (DMV)*, T. gondii, Herpesvirus* and *Brucella* spp. were routinely achieved on target tissues availables from Cases 1, 2 and 3, identified, respectively, in brain, lung, tonsils, lymph nodes, liver, spleen, kidney, bladder and blood for DMV, in brain, lymph nodes, liver, spleen, heart and muscle for *Toxoplasma*, in brain, lung, lymph nodes, spleen, kidney for *Herpesvirus* (integrated by liver for Case 3), and brain, lung, tonsils, lymph nodes, liver, spleen, kidney and blood for *Brucella* spp^26,27,49,50^.

For DMV and *Herpesvirus* assays, amplicons were directly sequenced using PCR primers on a 3130XL Genetic Analyzer (Thermo Fisher).Sequences were aligned using the SeqMan software (Lasergene package. DNASTAR Inc.) to obtain a consensus sequence and BLAST analysis was performed.

Virus isolation was carried out for *Morbillivirus* and *Herpesvirus* from PCR-positive frozen tissues, using, respectively, confluent monolayers of Vero Dog Slam cells^51^ and MDBK cells (Madin-Darby Bovine Kidney). All virus isolation attempts were unsuccessful.

The presence of anti-*T. gondii*, anti-*Brucella* spp. and anti-*Morbillivirus* antibodies was investigated in blood serum from Case 1 and in blood serum, humor aqueous and cerebrospinal fluid (CSF) from Case 2 and 3^25,52,53^. Specifically, anti-*Brucella* spp. antibodies were detected by rapid serum agglutination (S.A.R.), utilizing both *B. abortus* and *B. melitensis* as antigens, whereas anti-*T. gondii* antibodies were detected by an indirect immunofluorescence test (IFAT) and anti-*Morbillivirus* antibodies by virus neutralization assay (serum neutralization, SN), using the Onderstepoort strain of CDV^25^.

### Toxicology

Hexachlorobenzene (HCB), dichlorodiphenyltrichloroethane (DDT) and its metabolites (dichlorodiphenyldichloroethane (DDD) and dichlorodiphenyldichloroethylene (DDE)) and polychlorinated biphenyls (PCBs), as well as Extracted Organic Material percent (EOM%), were measured in blubber (Cases 2 and 3), according to the Environmental Protection Agency method 8081/8082, with revisions^54^. The OC levels are expressed both in ng/g dry weight and in ng/g lipid weight basis (l.w.) since variation in lipid content among organisms can affect contaminant concentrations.

Toxicological stress was evaluated using both a theoretical model^23^ and the toxicity threshold value set by Jepson *et al*.^21^ and Kannan *et al*.^22^. For Case 2 blubber residue levels were measured only on a lipid basis since this female specimen was experiencing a marked starvation, leading to an intense mobilization of her subcutaneous fat reserves. For this reason, the Extracted Organic Material (EOM) percentage was very similar in all the tissues and very low in the blubber (EOM% = 50.66), proving this metabolic imbalance.

### Accession codes

The Salmonella whole-genome sequences assembled have been deposited at DDBJ/ENA/GenBank under the accession no. QIBF00000000, QIBE00000000 and QIBD00000000; the version described in this paper is version 0.1 for all genomes.

## Data Availability

All data generated during and/or analysed during the current study are available from the corresponding author on reasonable request.
